# Aftermath

**DOI:** 10.3201/eid1401.079999

**Published:** 2008-01

**Authors:** George Held

It's not the storm itself—wind and rain lashing shore,
uprooting trees, toppling poles and dousing lights,
flooding cellars and roads, capsizing boats—
but the aftermath—the bright calm, the pair
of drowned cats crumpled against the picket fence,
the parlor of Izzy’s shack open for inspection,
the walls fallen flat on all sides, your own
roof filling the front yard, covering your car,
and your own twin daughters dazed by Nature’s
petulance—that makes you reconsider
your life and weigh your possessions and the cost
of putting down stakes too near the coast
as the globe warms, and storms grow worse.

